# Allele-specific expression of mutated in colorectal cancer (*MCC*) gene and alternative susceptibility to colorectal cancer in schizophrenia

**DOI:** 10.1038/srep26688

**Published:** 2016-05-26

**Authors:** Yang Wang, Yanfei Cao, Xiaoye Huang, Tao Yu, Zhiyun Wei, John McGrath, Fei Xu, Yan Bi, Xingwang Li, Fengping Yang, Weidong Li, Xia Zou, Zhihai Peng, Yanzeng Xiao, Yan Zhang, Lin He, Guang He

**Affiliations:** 1 Bio-X Institutes, Key Laboratory for the Genetics of Developmental and Neuropsychiatric Disorders (Ministry of Education), Shanghai Jiao Tong University, 1954 Huashan Road, Shanghai 200030, China; 2 Xin Hua Hospital Affiliated to Shanghai Jiao Tong University School of Medicine Shanghai 200092, China; 3 Institute for Nutritional Sciences, Shanghai Institutes of Biological Sciences, Chinese Academy of Sciences, 320 Yueyang Road, Shanghai 200031, China; 4 Department of Neurology, Center for Neurologic Diseases, Harvard Medical School, 77 Avenue Louis Pasteur, Boston, MA 02115, USA; 5 Queensland Centre for Mental Health Research, The Park Centre for Mental Health, Wacol, QLD 4076, Australia; 6 Queensland Brain Institute, University of Queensland, St Lucia, QLD 4072, Australia; 7 Ministry of Education Key Laboratory of Systems Biomedicine, Shanghai Center for Systems Biomedicine, Shanghai Jiao Tong University, 800 Dongchuan Road, Shanghai, 200240, China; 8 Department of General Surgery, The Affiliated Shanghai First People’s Hospital of Shanghai Jiao Tong University, 100 Hai Ning Road, Shanghai 200080, China; 9 Shanxi Cancer Hospital, 3 Zhi Gong Xin Street, Taiyuan 320013, China; 10 Institutes of Biomedical Sciences, Fudan University, 138 Yixueyuan Road, Shanghai 200032, China

## Abstract

Evidence has indicated that the incidence of colorectal cancer (CRC) among schizophrenia is lower than normal. To explore this potential protective effect, we employed an innovative strategy combining association study with allele-specific expression (ASE) analysis in *MCC* gene. We first genotyped four polymorphisms within *MCC* in 312 CRC patients, 270 schizophrenia patients and 270 controls. Using the MassArray technique, we performed ASE measurements in a second sample series consisting of 50 sporadic CRC patients, 50 schizophrenia patients and 52 controls. Rs2227947 showed significant differences between schizophrenia cases and controls, and haplotype analysis reported some significant discrepancies among these three subject groups. ASE values of rs2227948 and rs2227947 presented consistently differences between CRC (or schizophrenia) patients and controls. Of the three groups, highest frequencies of ASE in *MCC* were concordantly found in CRC group, whereas lowest frequencies of ASE were observed in schizophrenia group. Similar trends were confirmed in both haplotype frequencies and ASE frequencies (i.e. CRC > control > schizophrenia). We provide a first indication that *MCC* might confer alterative genetic susceptibility to CRC in individuals with schizophrenia promising to shed more light on the relationship between schizophrenia and cancer progression.

Since the Commissioners in Lunacy for England and Wales proposed in 1909 that a reduced incidence of cancer might be linked to schizophrenia^1^, considerable attention has been paid to this century-old enigma. A relatively low risk of colorectal cancer (CRC) has recently been reported among schizophrenia patients in comparison to the general population, and a similar relationship has also been validated in other types of cancer^2^,^3^. Schizophrenia patients tend to be exposed to poor diets, insufficient physical activity and heavy smoking, all of which are involved in tumorigenesis^4^. Although studies have indicated that genetic predisposition^5^ may partly explain the association between schizophrenia and cancer, so far only *p53* and *XRCC4* have been shown to be potential susceptibility genes for the possible protective mechanism against cancer progression^6^,^7^.

The mutated in colorectal cancer (*MCC*) gene is located on chromosome 5q21 and encodes a protein comprised of 829 amino acids that is highly conserved with orthologs across many species^8^. MCC protein is mainly localized in nuclear, cytoplasm and membrane-cytoskeletal components. Current reports suggest that MCC might be a key negative regulator in the cell cycle, implying an important role for MCC in cell growth and differentiation, and also indicate that MCC, as a β-catenin-interacting protein, could suppress Wnt/β-catenin signal transduction^9^. Mutations and loss of heterozygosity (LOH) in the *MCC* gene have previously been found to be relevant to colorectal cancer. Kohonen-Corish *et al*.^8^ have further suggested that promoter methylation of *MCC* is an early event in colon carcinogenesis leading to loss of RNA and protein expression of this gene. This evidence points strongly to *MCC* as a potential suppressor gene in CRC tumorigenesis. Additionally, Chromosome 5q21 has been suggested as having a susceptibility locus for schizophrenia in case-control studies, indicating that genes situated close to this region are associated with increased risk of schizophrenia^10^,^11^,^12^. Several reports^13^ have shown that the levels or localization of Wnt/β-catenin signaling components are altered in the cortex, subiculum and hippocampus of schizophrenia patients compared to unaffected individuals.

Allele-specific expression (ASE) in autosomal nonimprinted genes is currently regarded as a relatively common phenomenon^14^,^15^,^16^,^17^,^18^. The quantitative differences in gene expression levels are heritable and context-specific, and have been mentioned as an incomplete-penetrance marker involved in predisposition to both CRC and schizophrenia^18^,^19^,^20^. Moreover, ASE studies have explored an innovative method to uncover the respective contributions of cis-/trans-regulatory variation^21^,^22^. Other than in total abrogation of the expression of one allele, the allelic expression differences can be as small as 15–25%^19^ and are hard to detect. We therefore studied the ASE at the *MCC* locus among CRC patients, schizophrenia patients, and normal controls, using the MassArray technique which is based on the highly sensitive and accurate MALDI-TOF-Mass Spectrometry. By doing so, we aimed to confirm the hypothesis that the *MCC* gene may be involved in the genetically reduced susceptibility to colorectal cancer in patients with schizophrenia.

## Results

### Allele and genotype distributions of the genetic variants within *MCC* gene

Genotype distributions of all four polymorphisms showed no significant deviations from Hardy-Weinberg equilibrium in any of the three groups in the first sample series (312 CRC, colorectal cancer patients; 270 SZ, schizophrenia patients; 270 NC, normal controls). Data of all the markers assayed are summarized in Table 1, corresponding to each paired groups. We observed that the allele frequencies of rs2227947 presented statistically significant differences between SZ and NC (*p* = 0.005, *p* = 0.020 after the FDR correction) (Table 1). The T allele and TT genotype of rs2227947 were significantly more common in schizophrenia group compared to control group (allele, 71.6% versus 63.4%, OR 0.69, 95% CI 0.53–0.89; genotype, 51.5% versus 42.1%). Moreover, we further assessed the allele frequencies of the four markers in the second sample series, which consisted of 50 CRC, 50 SZ, and 52 NC (Table 2). We found a similar distribution of the allele frequencies in the two sample series, and also rs2227947 showed significant differences in the allele frequencies between SZ and NC in the second sample set (*p* = 0.039, *p* > 0.05 after the FDR correction) (Table 2).

### LD estimation and haplotype analysis

For each pair of markers, we recruited SHEsis to calculate LD between groups (SZ vs NC, CRC vs NC, and CRC vs SZ) (Table 3). All these four markers were observed to be in strong LD (D’ > 0.7), and we therefore estimated the haplotype distributions with these markers among the three independent groups. Haplotypes were omitted from analysis if the estimated haplotype probabilities were less than 3% in any of the three groups. Haplotypes which showed positive results were selected for presentation (Table 4). Moreover, haplotype analysis reported some significant global p values (Table 5). The haplotype, rs9122-rs2227948, was the most significant giving a global p = 0.0002 between CRC and SZ. As its frequency was greater in CRC than in SZ (or NC), the haplotype G-C-T (rs9122-rs2227948-rs2227947) was observed to be correlated with an increased odds ratio for CRC (CRC vs NC: *p* = 0.018, OR = 2.70, 95% CI 1.07–6.81; CRC vs SZ: *p* = 0.001, OR = 3.10, 95% CI 0.98–9.83), and the same situations were also observed in G-C (rs9122-rs2227948), G-T-C (rs9122-rs2112452-rs2227948), and G-T-C-T (rs9122-rs2112452-rs2227948-rs2227947).

### ASE measurements of individual SNPs and the *MCC* gene

**Figure 1:**
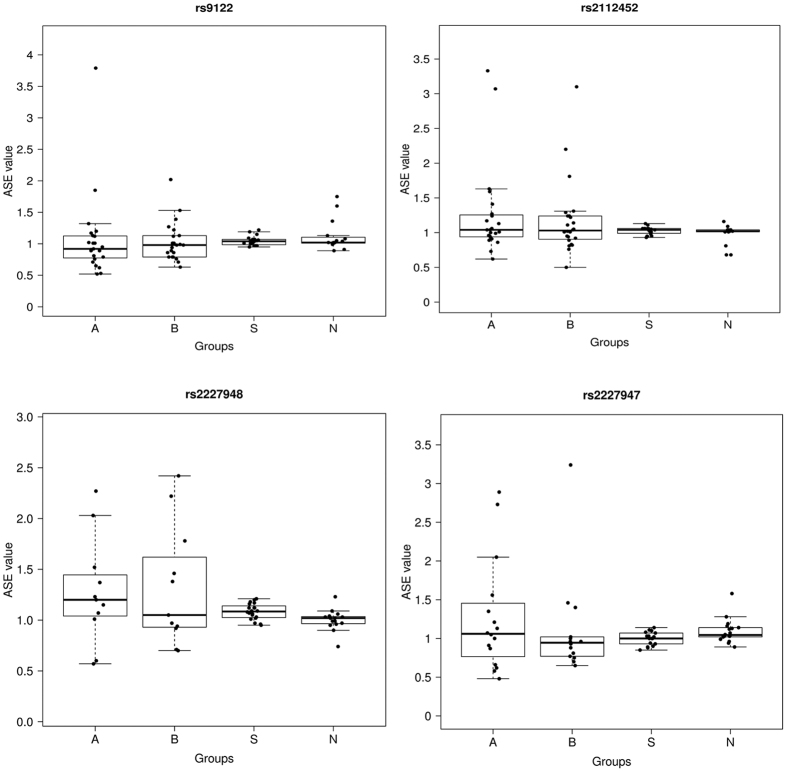
ASE distribution of single SNPs in 50 sporadic CRC patients, 50 schizophrenia patients and 52 normal controls analyzed by MassArray. ASE value was normalized using the formula: ASE value = cDNA (allele a expression/allele b expression)/gDNA (allele a expression/allele b expression). Allelic-expression ratios were represented by the measured peak area ratios in cDNA and gDNA. For each marker, ASE was detected in each heterozygous individual with two independent cDNA preparations each in duplicate so that ASE was calculated as the average of four different ratios. A, cancerous tissue; B, normal tissue from the same CRC patient; S, schizophrenia patients; N, normal controls.

**Figure 2:**
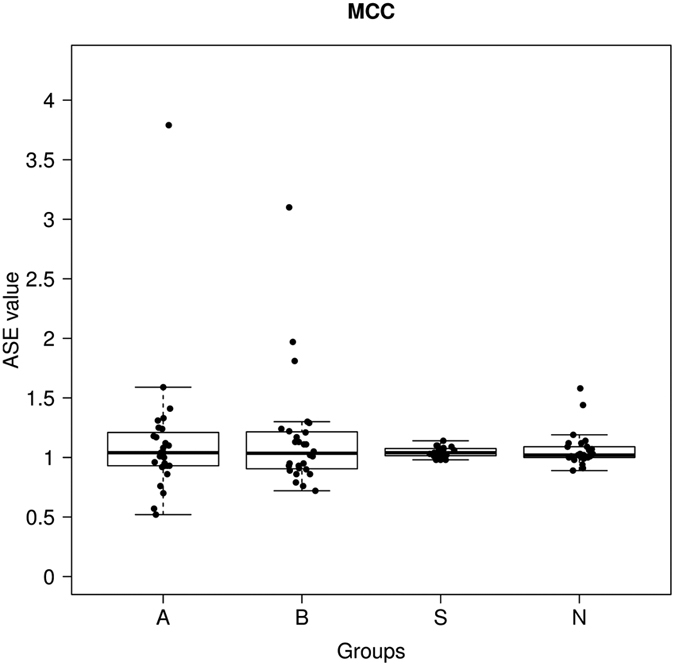
*MCC* ASE distribution in 50 sporadic CRC patients, 50 schizophrenia patients and 52 normal controls studied by MassArray. For the *MCC* gene, the final ASE value for each heterozygous sample was given as the average of the ASE values for all three SNPs assayed. A, cancerous tissue; B, normal tissue from the same CRC patient; S, schizophrenia patients; N, normal controls.

Using a second sample set comprised of 50 CRC patients (including cancerous and normal tissues), 50 schizophrenia patients and 52 normal controls, we studied ASE for both individual SNPs (rs9122, rs2112452, rs2227948 and rs2227947) and for the whole *MCC* gene (Figs 1 and 2; A, cancerous tissue; B, normal tissue from the same CRC patient; S, schizophrenia patient; N, normal control). The Wilcoxon rank sum test of rs2227948 showed positive results between A and N, and between S and N (A vs N, p = 0.0374; S vs N, p = 0.0112). For rs2227947, obvious differences were observed between B and N and between S and N (B vs N, p = 0.0228; S vs N, p = 0.0384) (Table 6).

### Assessment of ASE imbalances between the three subject groups

We further tested the overall diagnostic accuracy of varied cut-off points using the ROC analysis and Youden’s index as described previously^18^. Taking rs2227948 as an example, the final ASE cut-off point was achieved by maximizing Youden’s index at an ASE value of 1.2 (Table 7), which means a 17% difference in expression level between the two alleles. Consequently, all the heterozygous individuals were divided into ASE group (ASE ≥ 1.2 or ASE ≤ 0.83) and non-ASE group (0.83 < ASE < 1.2).

**Figure 3:**
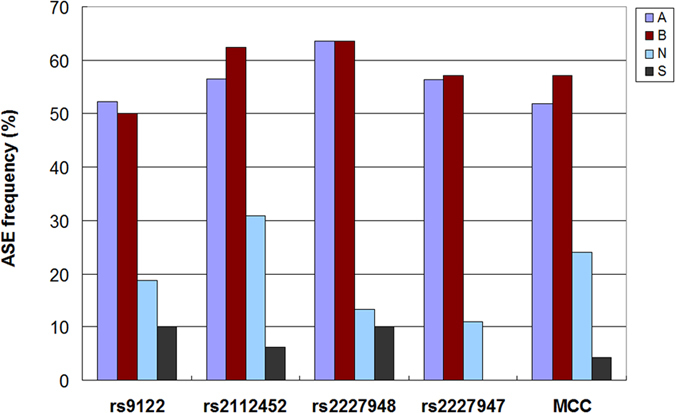
Comparison of ASE frequencies in CRC group, schizophrenia group and normal control group. Informative individuals were included in the analysis. For each group, ASE frequency = ASE count/Total count. A, cancerous tissue; B, normal tissue from the same CRC patient; S, schizophrenia patients; N, normal controls.

As all subjects involved in the present study belonged to either the ASE or non-ASE group, we compared ASE or non-ASE frequencies between CRC patients, schizophrenia patients and normal controls using the χ^2^ test or Fisher’s exact test (Table 8). With respect to informative individuals, *MCC* and all the individual markers except rs2112452 represented notable ASE imbalances (i.e. discrepancy in ASE frequencies) between CRC group and control (or schizophrenia) group, and significant discrepancy in ASE frequencies as to rs2112452 was found between CRC subjects and schizophrenia subjects. There were no statistically significant ASE imbalances between cancerous tissue and normal tissue from the same CRC patient, or between schizophrenia group and control group. When ASE assessment was performed in either informative subjects or all subjects (i.e. informative & non-informative subjects), the results corresponding to both the *MCC* gene and individual polymorphisms showed consistently higher ASE frequencies in CRC group (A or B) than in schizophrenia group (S) or control group (N). Although the differences in ASE frequencies were not statistically significant between schizophrenia group and control group, lower frequencies of ASE were consistently observed in schizophrenia group (S) compared to control group (N) (Fig. 3).

## Discussion

In an earlier study, we found that shared mechanisms underpinning cell cycle regulation and synaptic plasticity provide further support for the association between schizophrenia and colorectal cancer^23^. Evidence that *MCC* is heavily involved in the negative regulation of the cell cycle^9^ along with other evidence described above, led us to conduct a genetic analysis of the *MCC* gene among paired groups (CRC vs schizophrenia, CRC vs control, schizophrenia vs control), using a creative strategy combining association studies with ASE measurements.

We firstly conducted the association analysis by genotyping four SNPs, all of which were selected from the HapMap project database (http://www.hapmap.org) and dbSNP (http://www.ncbi.nlm.gov/SNP/). The data based on 852 Han Chinese subjects provides an indication that *MCC* might be involved in the alterative genetic susceptibility to CRC in individuals with schizophrenia. There were statistically significant differences of allele frequencies between SZ (schizophrenia patients) and NC (normal controls) at rs2227947 surviving the FDR correction. We observed that the T allele and TT genotype of rs2227947 were more frequent in SZ than in NC. Additionally, we observed a similar distribution of the allele frequencies by comparing the four markers between the first and second sample series (Tables 1 & 2). Since haplotypes constructed from closely located polymorphisms will typically increase the statistical power for association studies, we performed haplotype analysis in the four markers which presented strong linkage disequilibrium (*D*’ > 0.7). Our results indicated that rs9122-rs2227948 and rs9122-rs2112452-rs2227948 showed consistently significant differences in global frequencies between CRC (colorectal cancer patients) and NC or between CRC and SZ (Table 5). Besides, we found that the four haplotypes, including G-C (rs9122-rs2227948), G-T-C (rs9122-rs2112452-rs2227948), G-C-T (rs9122-rs2227948-rs2227947) and G-T-C-T (rs9122-rs2112452-rs2227948-rs2227947), were more common in CRC compared to SZ or NC (Table 4), in particular we observed that there was a trend in haplotype frequencies, i.e. CRC > NC > SZ, which implied that *MCC* might confer an alternative genetic susceptibility to CRC among SZ.

**Figure 4:**
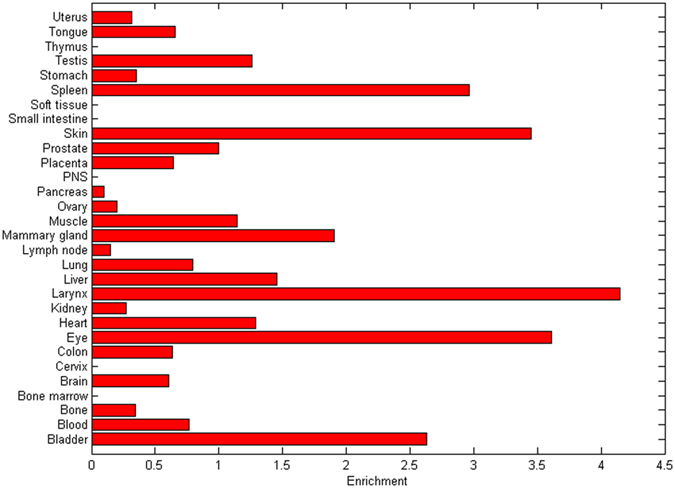
Tissue-specific expression analysis in the *MCC* gene. The expression level is normalized with tissue-library size. Each value for a gene in a tissue is a ratio of observed ESTs (Expressed Sequence Tags) to the expected one in this tissue. The expected number of ESTs is the product of total ESTs of the gene and the fraction of total ESTs in the tissue among all ESTs in 30 tissues.

**Figure 5:**
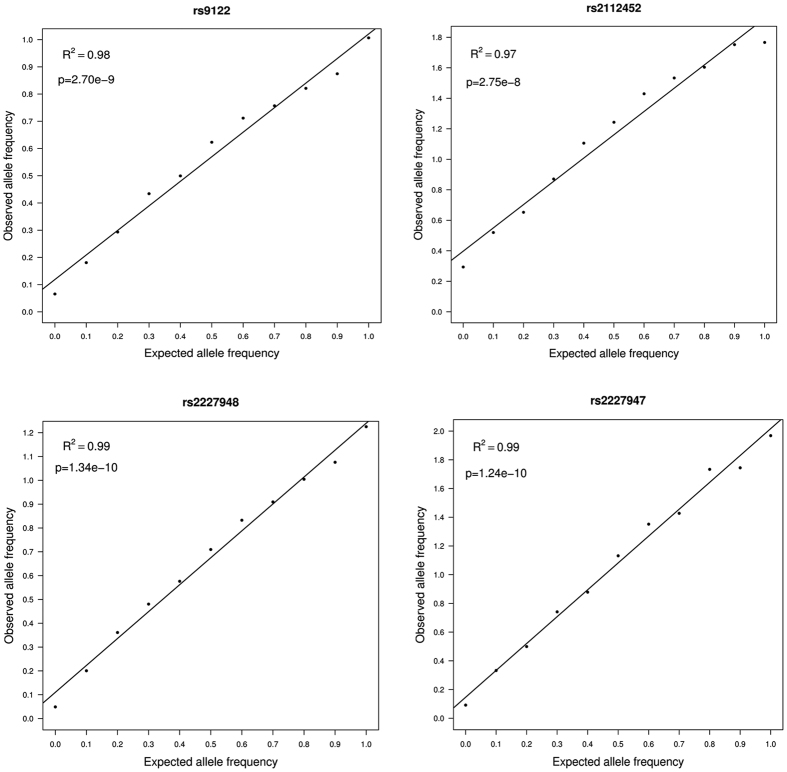
Evaluation of MassArray as a quantitative technique for allele-specific expression. For each SNP of the *MCC* gene, the two homozygous genomic DNAs as to the different alleles were mixed at known ratios (i.e. expected allele ratios). Observed allele ratios were obtained by the MassArray analysis of amplification products on each mixture. Pearson’s correlation test was involved in a further analysis.

On the basis that the extent of ASE for susceptibility genes might be tissue-dependent, we introduced the TiGER database to study tissue-specific gene expression levels. As shown in Fig. 4, no significant discrepancy was observed in *MCC* gene expression among brain tissue, colon tissue and peripheral blood, thus ensuring the feasibility of performing ASE detection with the blood samples of schizophrenia cases and normal controls. Taking a step further, we tried to test the accuracy of the MassArray platform in ASE analysis by mixing experiments. A concordant rectilinear correlation at each of the four SNPs (rs9122, rs2112452, rs2227948 and rs2227947) was found between the input of the two allelic variants and the resulting ratio (Fig. 5).

Our findings provide a unique perspective on genetic protection against CRC in patients with schizophrenia which might involve the *MCC* gene. For the heterozygous samples, ASE values of rs9122, rs2112452, rs2227948 and rs2227947 were in the range of 0.52–3.79, 0.5–3.33, 0.57–2.42, and 0.48–3.24 respectively (Fig. 1), and that of the *MCC* gene ranged from 0.52 to 3.79 (Fig. 2). Moreover, we were able to ensure the accuracy and repeatability of the ASE analysis based on our data by calculating each single ASE value as the average of four different ratios. We applied the Wilcoxon test to study the ASE degree of both individual SNPs and *MCC*, and found that the analysis of rs2227948 and rs2227947 reported concordantly positive results, implying a difference in ASE degree existed between CRC and NC, or between SZ and NC (Table 6). Both rs2227948 and rs2227947 are synonymous markers located in the coding region of the *MCC* gene, and it has been implied that synonymous SNPs may play a role in regulating mRNA secondary structure and stability, and exert downstream effects on the rate of translation, folding and post-translational modifications of nascent polypeptides^24^,^25^. On the other hand, ASE of synonymous SNPs has been documented in recent studies^26^,^27^, and since ASE is regarded as a molecular mechanism capable of modulating gene expression, the differences in the gene’s expression caused by ASE may further facilitate the impact of synonymous markers on gene functions, which therefore might contribute to the pathological conditions.

The genomic mechanisms causing ASE have been indicated to be a combined *cis* and *trans* effect^19^. In the present study, even if there were no statistically significant differences in the allele frequencies of rs2227948 between CRC (or schizophrenia) and control group, the discrepancies in the ASE values of rs2227948 were still observed between CRC (or schizophrenia) patients and controls, which might be due to a collaboration between genomic variants in *cis* and *trans* under pathological conditions. On the other hand, it has been demonstrated that ASE segregates not only with the phenotypes but also with the haplotypes covering all or part of the gene^19^. Actually we found that the haplotypes including G-C-T (rs9122-rs2227948-rs2227947) and G-T-C-T (rs9122-rs2112452-rs2227948-rs2227947), were more common in CRC group compared to schizophrenia or control group (Table 4), and similar trends were also observed in the ASE frequencies of both rs2227948 and rs2227947 (Table 8), further supporting the indication that genomic changes in *cis* are present.

Using ROC analysis and Youden’s index, we were able to determine the final ASE cut-off points, and thus all the participants were subjected to either ASE or non-ASE group^18^. With respect to *MCC* and all the single markers, ASE imbalances were notable between CRC and NC, or between CRC and SZ, and the frequencies of ASE were observed to be accordingly higher in CRC than in SZ or NC. Additionally, we found that the frequencies of ASE were consistently lower in SZ than in NC (Table 8). A trend similar to that observed in haplotype frequencies has also been found in ASE frequencies, i.e. CRC > NC > SZ (Fig. 3). In addition, our results showed no obvious ASE imbalances in cancerous tissues in comparison to the normal tissues from the same CRC patients. We further assessed the ASE imbalances by including non-informative individuals, and observed a similar situation as described above (Table 8). Of note, ASE analysis is more complex in the *MCC* gene compared to individual markers since it is more difficult to determine whether the individual is informative (heterozygous for a transcribed SNP) or not for the *MCC* gene compared to the individual SNP. When only informative individuals were taken into account, ASE of *MCC* occurred in 14/27 (51.9%) CRC subjects, 1/23 (4.3%) schizophrenia subjects, and 6/25 (24.0%) controls. If none of the non-informative individuals had ASE, the ASE frequencies as to *MCC* would be 14/50 (28.0%) in CRC group, 1/50 (2.0%) in schizophrenia group, and 6/52 (11.5%) in control group. Since not all individuals are informative regarding the *MCC* gene, recruiting more coding/UTR markers will be necessary to precisely assess the ASE frequencies of *MCC* among the three independent groups. Overall, the observations suggested that ASE might be involved in the complex relationship between schizophrenia and CRC tumorigenesis.

ASE analysis has provided an effective way to explore the impact of genetic variations on gene expression^28^. Heterozygous individuals were tested for allelic transcript levels that differed from each other, taking advantage of allelic ratios of gDNA as a control of 1:1 hybridization intensity^29^. Because the mRNA expression levels of two alleles of a heterozygous SNP are captured in the same cellular environment within the same sample, the alternative alleles serve as within-sample controls of each other, eliminating genetic background and environmental influences, as well as technical noise which affects both alleles equally, and making ASE assay more reliable for detecting significant differences^30^,^31^. Moreover, allele-specific expression analysis has been applied to identifying eQTLs (expression quantitative trait loci) which measures gene expression discrepancies among individuals with different genotypes, and can be integrated to facilitate the mapping of likely regulatory variants^29^.

A better understanding of the source of ASE imbalances will be necessary to clarify the mechanisms underlying phenotypic diversity and disease susceptibility. It is worth noting that as a common phenomenon found in humans, mice and maize, ASE exerts its impact on both normal development and many cellular processes, but if impaired, can lead to an increased risk of disease as indicated in the present study^32^,^33^,^34^,^35^. Though the allelic imbalances we mentioned here can be extremely subtle, they may have expansile/cumulative influences on the downstream signaling pathway. With the present limitations, still we are unable to clarify why allelic imbalances were naturally more common in CRC patients, and the cause of ASE remains unresolved, which may be due to cis/trans effect, or combined cis & trans effect^19^. Moreover, it remains elusive how small ASE imbalances could cause a phenotype.

There were some limitations in our study. Firstly, our sample size is relatively small. Because of the differences in genotype frequencies among CRC patients, schizophrenia patients and normal controls, and also because of the preponderance of homozygous individuals for the risk alleles in cases, it’s difficult to get a balanced representation of all genotypes and a desirable sample size. Secondly, the four SNPs we selected could not cover the whole region of MCC, thus additional replication studies using more SNPs in large Asian and non-Asian samples are needed. For obvious reasons, it is not possible to obtain gut tissue samples from SZ and NC, having to resort to use blood samples from schizophrenia subjects and controls.

We have provided a first indication that ASE as an inherited gene expression marker in *MCC* is more frequently found in CRC patients compared to schizophrenia patients and healthy controls, and the similar trends observed in both haplotype frequencies and ASE frequencies (i.e. CRC > NC > SZ) imply that *MCC* might be involved in the alternative genetic susceptibility to CRC among schizophrenia patients. Our present work and, hopefully, follow-up studies should together provide new insights into the question between schizophrenia and cancer progression (The abstract of our study was published at a scientific meeting). The advances in ASE analysis will no doubt shed more light on the critical etiopathogenesis of human complex diseases.

## Materials and Methods

### Participants

We used two CRC-schizophrenia-control series based on samples of Han Chinese origin. The first series, recruited for the association study, comprised 312 CRC patients (178 male and 134 female, age 61.23 ± 14.03 years), 270 schizophrenia patients (191 male and 79 female, age 57.25 ± 11.55 years) and 270 controls (145 male and 125 female, age 43.53 ± 7.94 years). The CRC cases were diagnosed in 1999–2007 in the Shanghai First People’s Hospital and the Shanxi People’s Hospital.

The second series was involved in the ASE analysis and consisted of 50 sporadic CRC cases (29 male and 21 female, age 62.02 ± 13.11 years), 50 schizophrenia cases (25 male and 25 female, age 53.00 ± 12.18 years), and 52 controls (27 male and 25 female, age 56.33 ± 14.23 years). Participants with sporadic CRC had all undergone curative surgery in the Ruijin Hospital, Shanghai. Cancerous and normal tissue (>10 cm) samples from the same CRC patient were immediately frozen in liquid nitrogen at the time of collection and were used for ASE analysis. Pathologic tumor staging was classified according to Duke’s criteria. For ASE measurements, peripheral blood samples were collected from schizophrenia patients and normal controls.

All the schizophrenia subjects were interviewed by two independent clinicians, and were diagnosed strictly according to the DSM-III-R criteria and hospital case notes. Normal controls with no personal history of cancer or psychiatric disorders were selected randomly from the population. All the schizophrenia patients and normal controls were from Shanghai. All participants signed the full informed consent. All experimental protocols were reviewed and approved by the Ethics Committee of the Human Genetics Center in Shanghai. All experiments were carried out in accordance with the standard procedures.

### Nucleic acid extraction and genotyping

Peripheral blood was drawn from schizophrenia cases and normal controls and was collected in EDTA tubes. Genomic DNA and RNA were isolated from peripheral blood using the phenol-chloroform method and QIAamp RNA blood mini kit (Qiagen, Valencia, CA) respectively. Extraction of DNA from tissue was performed by a proteinase K and standard phenol-chloroform procedure. Tissue samples for total RNA extraction were processed with TRIzol reagent (Invitrogen, Carlsbad, CA) and were then treated with DNase I (DNAfree^TM^, Ambion, Austin, TX) prior to cDNA synthesis using Superscript^TM^ III First-Strand Synthesis System for RT-PCR (Invitrogen, Carlsbad, CA).

All four genetic markers (rs9122, rs2112452, rs2227948 and rs2227947) were selected from the HapMap project database http://www.hapmap.org and dbSNP http://www.ncbi.nlm.nih.gov/SNP/ covering the MCC region. Only coding/UTR markers with higher minor allele frequency (MAF > 15%) in the Han Chinese population were recruited in the present study. Of the four markers, rs9122 and rs2112452 locate in the 3′-UTR, whereas rs2227948 and rs2227947 locate in the coding region and both of them are synonymous markers. We performed the standard 5 μl PCR using Taqman^®^ Universal PCR Master Mix (Applied Biosystems) reagent kit, and genotyped all the markers using the ABI 7900 DNA detection system (Applied Biosystems, Foster City, California).

### Assessment of tissue-specific expression and MassArray technique

We interrogated gene expression differences among brain tissue, colon tissue and peripheral blood taking advantage of TiGER (Tissue-specific Gene Expression and Regulation, http://bioinfo.wilmer.jhu.edu/tiger/) database^36^, which integrates tissue-specific gene expression profiles or expressed sequence tag data, cis-regulatory module data and combinatorial gene regulation data.

In addition, we used mixing experiments to assess the accuracy of MassArray technology in ASE measurements. DNA homozygous for one allele was first mixed in known proportions with DNA homozygous for the other allele^37^ and we were thus able to confirm whether the peak strengths of the two alleles were comparable at different proportions of the two alleles.

### Allele-specific expression (ASE) analysis

For informative individuals (i.e. heterozygotes), all four polymorphisms (rs9122, rs2112452, rs2227948 and rs2227947; MAF > 15%) within the *MCC* gene were subjected to MassArray detection in order to assess the expression discrepancies of the two alleles. We typed genomic DNA (gDNA) to identify heterozygotes for the markers described above since ASE to date can only be readily determined in subjects that are heterozygous for cSNPs or UTR SNPs. The distribution of heterozygous individuals corresponding to each SNP was presented in Table 9.

We fulfilled allele quantification for each targeted SNP, taking advantage of the MassArray platform which is based on MALDI-TOF-Mass Spectrometry^38^ and can capture sequence differences at the single nucleotide level when combined with iPLEX Gold reaction kit (Sequenom, Inc). The different mass signals corresponding to the two alleles can be detected by the highly accurate MALDI-TOF-based system. All the amplification and extension primers were designed by Assay Designer 4.0 (Sequenom, Inc).

The ASE ratio was measured as described previously^18^, and was normalized using the formula: ASE ratio = cDNA (allele a expression/allele b expression)/gDNA (allele a expression/allele b expression). Allelic-expression ratios were represented by the measured peak area ratios in cDNA and gDNA. For each SNP of each heterozygous subject, ASE detection was performed with two independent cDNA preparations each in duplicate so that ASE was calculated as the average of four different ratios^18^. For the *MCC* gene of each individual, the final ASE value was calculated as the average of the ASE values for all four polymorphisms.

### Statistical analysis

Allelic and genotypic distributions, Hardy-Weinberg equilibrium and Linkage disequilibrium (LD) were calculated on SHEsis (http://analysis2.bio-x.cn/myAnalysis.php)^39^, an online user-friendly platform which integrates efficient analysis tools particularly suited to association studies. We estimated LD by recruiting “D” as the standardized measure for all possible pairs of SNP loci. The program UNPHASED was used to perform haplotype analysis^40^. The odds ratios (OR) and 95% confidence intervals (CI) were calculated by using unconditional maximum likelihood estimation (Wald) method and normal approximation. In addition, significant *P* values of association analysis for multiplicity were subjected to a false discovery rate (FDR) controlling procedure^41^.

We used the Wilcoxon rank sum test to compare ASE values among the three independent groups, namely CRC subjects, schizophrenia subjects, and controls. 100,000 permutations were carried out to further compare the means of these three groups. Receiver operating characteristic (ROC) analysis and Youden’s index were employed to assess the overall diagnostic accuracy of different cut-off points^18^. Using R version 2.9.1, we performed the χ^2^ test or Fisher’s exact test (where expected cell count was less than 5) to compare ASE/non-ASE proportions between groups. Statistical significance level was set at *p* < 0.05.

## Additional Information

**How to cite this article**: Wang, Y. *et al*. Allele-specific expression of mutated in colorectal cancer (*MCC* ) gene and alternative susceptibility to colorectal cancer in schizophrenia. *Sci. Rep.* **6**, 26688; doi: 10.1038/srep26688 (2016).

## Figures and Tables

**Table 1:** Allele and genotype frequencies of 4 SNPs.

Groups	SNP ID	genotype frequency(%)			H-W check p value	*p* value*	allele frequency(%)		X^2^	*p* value*	Odds Ratio (95%CI)
SZ vs NC	rs9122	AA	AG	GG			A	G			
	SZ	30 (11.7)	104 (40.5)	123 (47.9)	0.271	0.160	164 (31.9)	350 (68.1)	3.609	0.057	1.28 (0.99–1.66)
	NC	38 (14.8)	117 (45.5)	102 (39.7)	0.639		193 (37.5)	321 (62.5)			
	rs2112452	CC	CT	TT			C	T			
	SZ	3 (1.3)	70 (29.3)	166 (69.5)	0.141	0.350	76 (15.9)	402 (84.1)	1.883	0.170	1.26 (0.90–1.76)
	NC	5 (2.0)	84 (34.4)	155 (63.5)	0.095		94 (19.3)	394 (80.7)			
	rs2227948	CC	CT	TT			C	T			
	SZ	33 (12.4)	107 (40.1)	127 (47.6)	0.164	0.115	173 (32.4)	361 (67.6)	3.583	0.058	1.27 (0.99–1.64)
	NC	39 (14.5)	126 (46.8)	104 (38.7)	0.933		204 (37.9)	334 (62.1)			
	rs2227947	CC	CT	TT			C	T			
	SZ	22 (8.4)	105 (40.1)	135 (51.5)	0.804	**0.019**	149 (28.4)	375 (71.6)	7.927	**0.005**	0.69 (0.53–0.89)
	NC	40 (15.3)	111 (42.5)	110 (42.1)	0.177		191 (36.6)	331 (63.4)			
CRC vs NC	rs9122	AA	AG	GG			A	G			
	CRC	36 (12.8)	110 (39.1)	135 (48.0)	0.075	0.149	182 (32.4)	380 (67.6)	3.153	0.076	1.26 (0.98–1.61)
	NC	38 (14.8)	117 (45.5)	102 (39.7)	0.639		193 (37.5)	321 (62.5)			
	rs2112452	CC	CT	TT			C	T			
	CRC	11 (3.8)	76 (25.9)	206 (70.3)	0.239	0.066	98 (16.7)	488 (83.3)	1.169	0.280	1.19 (0.87–1.62)
	NC	5 (2.0)	84 (34.4)	155 (63.5)	0.095		94 (19.3)	394 (80.7)			
	rs2227948	CC	CT	TT			C	T			
	CRC	43 (14.4)	135 (45.3)	120 (40.3)	0.615	0.921	221 (37.1)	375 (62.9)	0.085	0.771	1.04 (0.81–1.32)
	NC	39 (14.5)	126 (46.8)	104 (38.7)	0.933		204 (37.9)	334 (62.1)			
	rs2227947	CC	CT	TT			C	T			
	CRC	34 (11.8)	117 (40.5)	138 (47.8)	0.235	0.303	185 (32.0)	393 (68.0)	2.561	0.110	0.82 (0.64–1.05)
	NC	40 (15.3)	111 (42.5)	110 (42.1)	0.177		191 (36.6)	331 (63.4)			
CRC vs SZ	rs9122	AA	AG	GG			A	G			
	CRC	36 (12.8)	110 (39.1)	135 (48.0)	0.075	0.904	182 (32.4)	380 (67.6)	0.028	0.867	0.98 (0.76–1.26)
	SZ	30 (11.7)	104 (40.5)	123 (47.9)	0.271		164 (31.9)	350 (68.1)			
	rs2112452	CC	CT	TT			C	T			
	CRC	11 (3.8)	76 (25.9)	206 (70.3)	0.239	0.159	98 (16.7)	488 (83.3)	0.131	0.718	0.94 (0.68–1.30)
	SZ	3 (1.3)	70 (29.3)	166 (69.5)	0.141		76 (15.9)	402 (84.1)			
	rs2227948	CC	CT	TT			C	T			
	CRC	43 (14.4)	135 (45.3)	120 (40.3)	0.615	0.216	221 (37.1)	375 (62.9)	2.720	0.099	0.81 (0.64–1.04)
	SZ	33 (12.4)	107 (40.1)	127 (47.6)	0.164		173 (32.4)	361 (67.6)			
	rs2227947	CC	CT	TT			C	T			
	CRC	34 (11.8)	117 (40.5)	138 (47.8)	0.235	0.380	185 (32.0)	393 (68.0)	1.660	0.198	1.18 (0.92–1.53)
	SZ	22 (8.4)	105 (40.1)	135 (51.5)	0.804		149 (28.4)	375 (71.6)			

^*^Pearson’s *p* value, SNP = single nucleotide polymorphism, CI = confidence interval, CRC = colorectal cancer patients, SZ = schizophrenia patients, NC=normal controls.

**Table 2:** Allele frequencies of 4 SNPs in the second series

Groups	SNP ID	allele frequency(%)		X^2^	*p* value*	Odds Ratio (95%CI)
SZ vs NC	rs9122	A	G			
	SZ	30 (30.0)	70 (70.0)	0.496	0.481	0.81 (0.45–1.46)
	NC	36 (34.6)	68 (65.4)			
	rs2112452	C	T			
	SZ	18 (18.0)	82 (82.0)	0.159	0.691	0.87 (0.43–1.75)
	NC	21 (20.2)	83 (79.8)			
	rs2227948	C	T			
	SZ	28 (28.0)	72 (72.0)	2.972	0.085	0.60 (0.33–1.08)
	NC	41 (39.4)	63 (60.6)			
	rs2227947	C	T			
	SZ	25 (25.0)	75 (75.0)	4.255	**0.039**	0.53 (0.29–0.97)
	NC	40 (38.5)	64 (61.5)			
CRC vs NC	rs9122	A	G			
	CRC	33 (33.0)	67 (67.0)	0.059	0.807	0.93 (0.52–1.66)
	NC	36 (34.6)	68 (65.4)			
	rs2112452	C	T			
	CRC	25 (25.0)	75 (75.0)	0.675	0.411	1.32 (0.68–2.55)
	NC	21 (20.2)	83 (79.8)			
	rs2227948	C	T			
	CRC	39 (39.0)	61 (61.0)	0.004	0.951	0.98 (0.56–1.72)
	NC	41 (39.4)	63 (60.6)			
	rs2227947	C	T			
	CRC	32 (32.7)	66 (67.4)	0.776	0.389	0.78 (0.44–1.38)
	NC	40 (38.5)	64 (61.5)			
CRC vs SZ	rs9122	A	G			
	CRC	33 (33.0)	67 (67.0)	0.209	0.648	1.15 (0.63–2.09)
	SZ	30 (30.0)	70 (70.0)			
	rs2112452	C	T			
	CRC	25 (25.0)	75 (75.0)	1.452	0.228	1.52 (0.77–3.00)
	SZ	18 (18.0)	82 (82.0)			
	rs2227948	C	T			
	CRC	39 (39.0)	61 (61.0)	2.716	0.099	1.64 (0.91–2.98)
	SZ	28 (28.0)	72 (72.0)			
	rs2227947	C	T			
	CRC	32 (32.7)	66 (67.4)	1.414	0.234	1.45 (0.78–2.70)
	SZ	25 (25.0)	75 (75.0)			

*Pearson’s *p* value, SNP = single nucleotide polymorphism, CI = confidence interval,

CRC = colorectal cancer patients, SZ = schizophrenia patients, NC=normal controls.

**Table 3:** Estimation of linkage disequilibrium between the 4 SNPs.

Groups		rs9122	rs2112452	rs2227948	rs2227947
SZ vs NC	rs9122		**0.97**	**0.94**	**0.76**
	rs2112452	0.39		**0.97**	**0.71**
	rs2227948	0.87	0.37		**0.79**
	rs2227947	0.53	0.22	0.57	
CRC vs NC	rs9122		**0.97**	**0.92**	**0.77**
	rs2112452	0.39		**0.94**	**0.77**
	rs2227948	0.76	0.32		**0.81**
	rs2227947	0.56	0.26	0.57	
CRC vs SZ	rs9122		**0.94**	**0.95**	**0.80**
	rs2112452	0.36		**0.92**	**0.81**
	rs2227948	0.79	0.30		**0.83**
	rs2227947	0.57	0.30	0.57	

For each pair of SNPs, *D*’ > 0.7 are shown in boldface,

D’ values are shown above and r^2^ values below the diagoal,

SNP = single nucleotide polymorphism, CRC = colorectal cancer, SZ = schizophrenia,

NC = normal control.

**Table 4:**
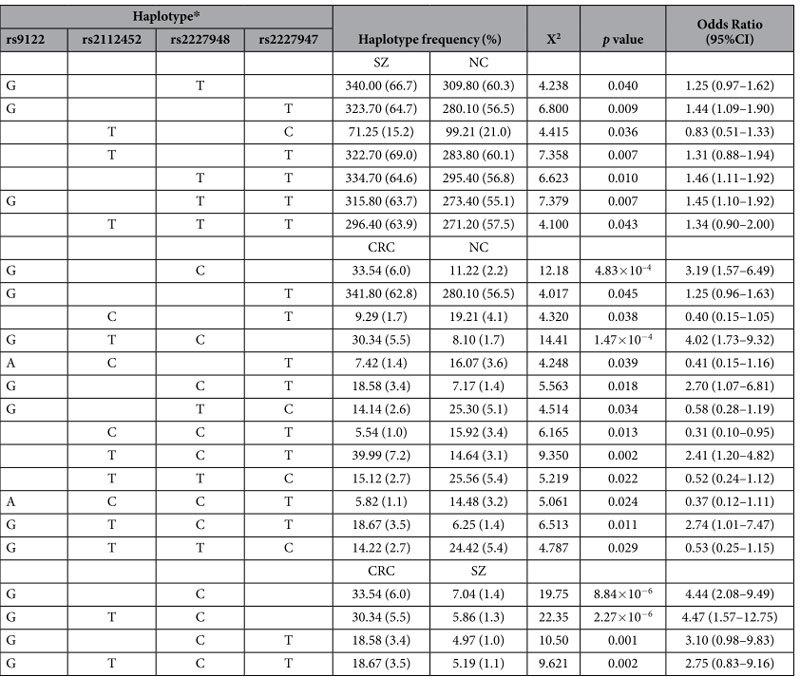
Estimated haplotype frequencies and association significance.

*Haplotypes were omitted from analysis if the estimated haplotype probabilities were less than 3%

CI = confidence interval, CRC = colorectal cancer patients, SZ = schizophrenia patients, NC=normal controls.

**Table 5:** Global p values of estimated haplotypes of the 4 SNPs within MCC.

Groups	Haplotype	Global *p* value*
SZ vs NC	rs2112452 - rs2227947	0.0435
	rs2227948 - rs2227947	0.0395
CRC vs NC	rs9122 - rs2227948	0.0030
	rs2112452 - rs2227947	0.0434
	rs9122 - rs2112452 - rs2227948	0.0060
	rs9122 - rs2227948 - rs2227947	0.0362
	rs2112452 - rs2227948 - rs2227947	0.0027
	rs9122 - rs2112452 - rs2227948 - rs2227947	0.0144
CRC vs SZ	rs9122 - rs2227948	0.0002
	rs9122 - rs2112452 - rs2227948	0.0020
	rs9122 - rs2227948 - rs2227947	0.0323

*Pearson’s *p* value, statistical significance set at *p* < 0.05, SNP = single nucleotide polymorphism.

CRC = colorectal cancer patients, SZ = schizophrenia patients, NC = normal controls.

**Table 6:** ASE values of individual SNPs and the *MCC* gene using Wilcoxon sum test.

Gene/SNP ID	Group	Wilcoxon *P* value	100,000 permutations
rs9122	A vs N	0.0555	0.8500
	B vs N	0.0564	0.3637
	S vs N	0.7827	0.2265
	A vs S	0.0682	0.9500
	B vs S	0.1577	0.8600
	A vs B	0.6323	0.8615
rs2112452	A vs N	0.3916	0.1394
	B vs N	0.3645	0.1956
	S vs N	0.4145	0.1847
	A vs S	0.8487	0.2178
	B vs S	0.9076	0.3204
	A vs B	0.8043	0.6677
rs2227948	A vs N	**0.0374**	0.0523
	B vs N	0.5150	0.0445
	S vs N	**0.0112**	**0.0113**
	A vs S	0.1473	0.1128
	B vs S	0.9757	0.0721
	A vs B	0.9487	0.8432
rs2227947	A vs N	0.9389	0.3574
	B vs N	**0.0228**	0.9643
	S vs N	**0.0384**	**0.0271**
	A vs S	0.5041	0.1617
	B vs S	0.1960	0.7039
	A vs B	0.5049	0.5679
*MCC*	A vs N	0.8168	0.7539
	B vs N	0.8701	0.4557
	S vs N	0.7781	0.4661
	A vs S	0.8356	0.6333
	B vs S	0.9813	0.3136
	A vs B	0.9767	0.9042

SNP = single nucleotide polymorphism

A = Cancerous tissue, B = Normal tissue from the same CRC patient,

S = Schizophrenia patients, N = Normal controls.

**Table 7:** Sensitivity, specificity and Youden’s index for different ASE cut-off values (rs2227948).

ASE cut-off	Sensitivity	Specificity	Youden’s index
1.0	1	0	0
1.1	0.82	0.60	0.42
**1.2**	**0.64**	**1**	**0.64**
1.3	0.55	1	0.55
1.4	0.45	1	0.45
1.6	0.36	1	0.36
1.7	0.27	1	0.27
1.9	0.18	1	0.18
2.2	0.09	1	0.09
3.3	0	1	0

**Table 8:** ASE frequencies of individual SNPs and the MCC gene among CRC patients, schizophrenia patients and normal controls.

Gene/SNP ID	ASE cut-off	Subjects	ASE/non-ASE counts* (ASE/non-ASE counts**)	ASE frequency*, % (ASE frequency**, %)	Group	P value* (χ^2^ or Fisher)	P value** (χ^2^ or Fisher)
rs9122	≥1.18 or ≤0.85	A	12/11 (12/38)	52.2 (24.0)	A vs N	0.0348	0.0094
		B	11/11 (11/39)	50.0 (22.0)	B vs N	0.0486	0.0173
		S	2/18 (2/48)	10.0 (4.0)	S vs N	0.6371	1
		N	3/13 (3/49)	18.8 (5.8)	A vs S	0.0032	0.004
					B vs S	0.0051	0.0074
					A vs B	0.8841	0.8122
rs2112452	≥1.12 or ≤0.90	A	13/10 (13/37)	56.5 (26.0)	A vs N	0.1371	0.0131
		B	15/9 (15/35)	62.5 (30.0)	B vs N	0.0653	0.0038
		S	1/15 (1/49)	6.3 (2.0)	S vs N	0.144	0.3629
		N	4/9 (4/48)	30.8 (7.7)	A vs S	0.0013	0.0005
					B vs S	0.0004	0.0001
					A vs B	0.6763	0.656
rs2227948	≥1.20 or ≤0.84	A	7/4 (7/43)	63.6 (14.0)	A vs N	0.0135	0.071
		B	7/4 (7/43)	63.6 (14.0)	B vs N	0.0135	0.071
		S	2/18 (2/48)	10.0 (4.0)	S vs N	1	1
		N	2/13 (2/50)	13.3 (3.8)	A vs S	0.0033	0.0806
					B vs S	0.0033	0.0806
					A vs B	1	1
rs2227947	≥1.23 or ≤0.81	A	9/7 (9/41)	56.3 (18.0)	A vs N	0.005	0.0212
		B	8/6 (8/42)	57.1 (16.0)	B vs N	0.0084	0.0391
		S	0/17 (0/50)	0.0 (0.0)	S vs N	0.4857	0.4952
		N	2/16 (2/50)	11.1 (3.8)	A vs S	0.0003	0.0017
					B vs S	0.0004	0.0032
					A vs B	0.9607	0.7901
*MCC*	≥1.12 or ≤0.89	A	14/13 (14/36)	51.9 (28.0)	A vs N	0.0391	0.0363
		B	16/12 (16/34)	57.1 (32.0)	B vs N	0.0145	0.012
		S	1/22 (1/49)	4.3 (2.0)	S vs N	0.0995	0.1125
		N	6/19 (6/46)	24.0 (11.5)	A vs S	0.0003	0.0003
					B vs S	0.0001	0.0007
					A vs B	0.6936	0.6625

SNP = single nucleotide polymorphism;

*informative individuals were included in the analysis; **both informative and non-informative individuals were included in the analysis;

A = Cancerous tissue, B = Normal tissue from the same CRC patient, S = Schizophrenia patients, N = Normal controls.

ASE frequency = ASE count/Total count.

**Table 9:** Distribution of informative individuals.

SNP ID	Subjects	Informative individuals Counts
rs9122	A	23
	B	22
	S	20
	N	16
rs2112452	A	23
	B	24
	S	16
	N	13
rs2227948	A	11
	B	11
	S	20
	N	15
rs2227947	A	16
	B	14
	S	17
	N	18

SNP = single nucleotide polymorphism,

informative individuals = heterozygous individuals.
